# Complete blood count–derived inflammatory parameters in pediatric idiopathic uveitis

**DOI:** 10.1186/s12886-026-05343-1

**Published:** 2026-09-15

**Authors:** Sema Nur Taşkın, Şeyda Doğantan, Dilbade Yıldız Ekici

**Affiliations:** 1 Division of Pediatric Rheumatology, Department of Pediatrics, Diyarbakır Children’s Hospital, Diyarbakır, Türkiye; 2 Division of Pediatric Rheumatology, Department of Pediatrics, University of Health Sciences Türkiye, Mersin City Hospital, Mersin, Türkiye; 3 Department of Ophthalmology, Diyarbakır Gazi Yaşargil Training and Research Hospital, Diyarbakır, Türkiye

**Keywords:** Pediatric uveitis, Idiopathic uveitis, Complete blood count, Inflammatory biomarkers, Disease activity, Mean platelet volume, Red cell distribution width, Platelet-to-monocyte ratio, Subclinical inflammation

## Abstract

**Background:**

Pediatric idiopathic uveitis is a potentially vision-threatening inflammatory condition with limited available laboratory biomarkers for disease monitoring. Complete blood count (CBC)–derived inflammatory parameters have emerged as inexpensive and accessible markers of systemic inflammation in various inflammatory diseases. This study aimed to investigate the potential role of CBC-derived inflammatory parameters in pediatric idiopathic uveitis and their association with disease activity.

**Methods:**

This retrospective study included 40 children with idiopathic uveitis and 44 age-matched healthy controls. Comparisons were performed between patients with active uveitis and healthy controls, between patients in remission and healthy controls, and between the active and remission phases of the same patients. The same patients were evaluated during both active disease and remission periods. Patients with elevated C-reactive protein (CRP) levels, acute infection, systemic disease, or corticosteroid use at the time of blood sampling were excluded. Hematological parameters including white blood cell count (WBC), platelet count (PLT), mean platelet volume (MPV), red cell distribution width (RDW), neutrophil count, lymphocyte count, neutrophil-to-lymphocyte ratio (NLR), platelet-to-lymphocyte ratio (PLR), and platelet-to-monocyte ratio (PMR) were analyzed.

**Results:**

A total of 40 children with idiopathic uveitis, all of whom had normal CRP levels, and 44 age- and sex-matched healthy controls were included. Compared with healthy controls, patients during the active phase had significantly higher WBC, RDW, PLT, MPV, neutrophil count, lymphocyte count, and PMR values (all *p* < 0.05). During remission, RDW, PLT, MPV, and lymphocyte counts remained significantly elevated, whereas NLR values were lower than those of healthy controls, with borderline statistical significance (*p* = 0.049). In paired comparisons, WBC, PLT, neutrophil count, and PMR values were significantly higher during active disease than during remission, whereas MPV values were significantly higher during remission (all *p* < 0.05).

**Conclusions:**

CBC-derived inflammatory parameters, particularly RDW, platelet-related indices, and PMR, may reflect inflammatory activity in pediatric idiopathic uveitis despite normal CRP levels. Persistent alterations during remission may indicate ongoing subclinical inflammation. These inexpensive and readily available parameters may serve as complementary biomarkers in disease evaluation and follow-up.

**Supplementary Information:**

The online version contains supplementary material available at https://doi.org/10.1186/s12886-026-05343-1.

## Introduction

Uveitis is an intraocular inflammatory disorder that may lead to serious vision-threatening complications, including cataract, glaucoma, macular edema, band keratopathy, and permanent visual loss if not diagnosed and treated appropriately. Although uveitis may occur as an isolated entity, as in idiopathic cases, it may also develop secondary to infectious causes, autoimmune and autoinflammatory diseases, trauma, malignancies, or various systemic syndromes [1, 2]. Although relatively uncommon in childhood, pediatric uveitis is associated with a more chronic and insidious course than in adults, resulting in delayed diagnosis and a higher risk of permanent ocular complications [2, 3].

Idiopathic uveitis represents one of the most common forms of pediatric non-infectious uveitis after exclusion of infectious and systemic inflammatory causes. In contrast to juvenile idiopathic arthritis-associated uveitis, for which several clinical risk factors have been established, reliable laboratory biomarkers for the diagnosis and monitoring of idiopathic pediatric uveitis remain limited [2, 4].

The management of pediatric uveitis generally requires a long-term, stepwise, and multidisciplinary treatment approach involving pediatric rheumatologists and ophthalmologists. Early diagnosis and timely initiation of treatment are particularly important in children, as uncontrolled inflammation may adversely affect both visual prognosis and quality of life [2, 3]. Despite this unmet clinical need, no specific laboratory biomarker has been established for the diagnosis or routine monitoring of idiopathic pediatric uveitis. In recent years, complete blood count-derived inflammatory markers such as neutrophil-to-lymphocyte ratio (NLR), platelet-to-lymphocyte ratio (PLR), platelet-to-monocyte ratio (PMR), mean platelet volume (MPV), and red cell distribution width (RDW) have gained increasing importance in the assessment of systemic inflammation. These parameters have been investigated in various inflammatory diseases because they are inexpensive, readily accessible, and routinely available in daily clinical practice. However, data regarding the potential role of these biomarkers in childhood uveitis, particularly idiopathic uveitis, remain limited [5, 6].

Therefore, in the present study, we aimed to investigate whether peripheral blood parameters routinely evaluated in children with idiopathic uveitis could help distinguish patients from healthy controls and reflect disease activation during follow-up.

## Methods

This retrospective study included 40 children diagnosed with idiopathic uveitis who were followed between February 2022 and January 2023. Patients were evaluated in the pediatric rheumatology and ophthalmology outpatient clinics of our institutions. Clinical assessments and follow-up evaluations were performed collaboratively by pediatric rheumatologists and ophthalmologists through a multidisciplinary approach. The study was approved by the local ethics committee (04 August 2023; decision no. 500) and was conducted in accordance with the principles of the Declaration of Helsinki.

Children aged 2–18 years who had been diagnosed with uveitis by an ophthalmologist were comprehensively evaluated for infectious, rheumatologic, neurologic, and other systemic conditions that could potentially cause uveitis. Patients in whom these etiologies were excluded were classified as having idiopathic uveitis and were subsequently enrolled in the study. None of the patients were receiving either systemic or topical corticosteroid therapy at the time of blood sampling.

The anatomical classification of uveitis (anterior, intermediate, or panuveitis) was determined according to the Standardization of Uveitis Nomenclature (SUN) criteria based on ophthalmologic examination findings [7]. Laterality was recorded as unilateral or bilateral according to the presence of ocular involvement at the time of diagnosis.

The same cohort of 40 patients was assessed during two distinct clinical periods according to ophthalmologic examination findings: the active disease period and the remission period. Active disease was defined as the presence of ongoing intraocular inflammation, whereas remission was defined as inactive disease for at least three months. A total of 44 age- and sex-matched healthy children with no history of ocular or systemic disease were included as the control group.

C-reactive protein (CRP) levels were within normal limits in all participants in the active period, remission period, and control groups. Patients with evidence of acute infection at the time of evaluation, elevated CRP levels, concomitant systemic disease, or significant abnormalities in hematologic or biochemical parameters were excluded from the study.

Venous blood samples were collected under standardized conditions into ethylenediaminetetraacetic acid (EDTA) tubes and analyzed within one hour of sampling. Complete blood count parameters, including white blood cell count (WBC), hemoglobin level, platelet count (PLT), MPV, RDW, neutrophil count, lymphocyte count, and monocyte count, were recorded. In addition, NLR, PLR, and PMR were calculated. The measurement units and reference ranges routinely used in our institutional laboratory were as follows: WBC, 4–10 × 10³/µL; hemoglobin, 11–16 g/dL; platelet count, 100–400 × 10³/µL; MPV, 6.5–12 fL; RDW, 11–16%; neutrophil count, 2–7 × 10³/µL; lymphocyte count, 0.8–4 × 10³/µL; monocyte count, 0.12–1.2 × 10³/µL; and CRP, < 5 mg/L. As calculated indices, NLR, PLR, and PMR did not have predefined institutional reference ranges.

Statistical analyses were performed using IBM SPSS Statistics version 20.0 (IBM Corp., Chicago, IL, USA). Continuous variables were expressed as mean ± standard deviation or median (interquartile range), as appropriate. The normality of data distribution was assessed using the Shapiro–Wilk test. Comparisons were performed between patients with active uveitis and healthy controls, between patients in remission and healthy controls, and between the active and remission periods of the same patients. Independent-group comparisons were analyzed using Student’s *t*-test or the Mann–Whitney *U* test, as appropriate, whereas paired comparisons were analyzed using the paired Student’s *t*-test or Wilcoxon signed-rank test, as appropriate. A p value < 0.05 was considered statistically significant.

## Results

A total of 40 children with idiopathic uveitis (mean age, 12.71 ± 2.96 years; age range, 5.5–17.5 years; 20 females [50.0%]) and 44 age- and sex-matched healthy controls (mean age, 12.56 ± 4.04 years; age range, 3–17 years; 22 females [50.0%]) were included in the study. The same 40 patients were evaluated during both the active disease period and remission period. The demographic and clinical characteristics of the idiopathic uveitis cohort are summarized in Table 1.

**Table 1:** Demographic and clinical characteristics of children with idiopathic uveitis (*n* = 40)

Variable	Idiopathic uveitis (*n* = 40)
Age (years), mean ± SD	12.71 ± 2.96
Female sex, n (%)	20 (50.0)
Male sex, n (%)	20 (50.0)
Anterior uveitis, n (%)	10 (25.0)
Intermediate uveitis, n (%)	14 (35.0)
Panuveitis, n (%)	16 (40.0)
Bilateral involvement, n (%)	39 (97.5)
Unilateral involvement, n (%)	1 (2.5)

Values are presented as mean ± SD or n (%)

When patients in the active phase were compared with healthy controls, WBC, RDW, PLT, MPV, neutrophil count, lymphocyte count, and PMR were significantly higher in the active uveitis group. No significant differences were observed in Hb, monocyte count, NLR, or PLR (Table 2)

**Table 2:** Comparison of hematological parameters between patients during active uveitis and healthy controls

Parameter	Active uveitis period	Control group	*p* value
WBC (×10³/µL)	9.23 (7.22–10.78)	6.90 (5.64–9.01)	**0.001**
Hb (g/dL)	13.20 ± 0.84	13.41 ± 1.01	0.311
RDW (%)	13.75 ± 1.25	12.79 ± 1.46	**0.002**
PLT (×10³/µL)	346.40 ± 71.09	276.82 ± 54.24	**< 0.001**
MPV (fL)	9.75 ± 1.32	7.33 ± 0.90	**< 0.001**
Neutrophil (×10³/µL)	4.86 (3.61–5.89)	3.59 (2.92–5.13)	**0.019**
Lymphocyte (×10³/µL)	3.11 (2.32–3.65)	2.28 (1.99–2.77)	**0.003**
Monocyte (×10³/µL)	0.53 (0.35–0.64)	0.47 (0.41–0.56)	0.428
NLR	1.49 (1.08–2.07)	1.53 (1.32–2.13)	0.444
PLR	110.78 (94.67–133.57)	112.18 (92.15–148.23)	0.982
PMR	664.26 (565.53–879.22)	563.63 (418.70–734.91)	**0.018**

*Values are presented as mean ± standard deviation for normally distributed variables and median (interquartile range) for non-normally distributed variables. Comparisons between independent groups were performed using Student’s t-test or the Mann–Whitney U test, as appropriate.Abbreviations: WBC, white blood cell count; Hb, hemoglobin; RDW, red cell distribution width; PLT, platelet count; MPV, mean platelet volume; NLR, neutrophil-to-lymphocyte ratio; PLR, platelet-to-lymphocyte ratio; PMR, platelet-to-monocyte ratio

When patients in the remission phase were compared with healthy controls, RDW, PLT, MPV, and lymphocyte counts were significantly higher, whereas NLR values were lower, with borderline statistical significance (*p* = 0.049). No significant differences were observed in WBC, hemoglobin level, neutrophil count, monocyte count, PLR, or PMR (Table 3)

**Table 3:** Comparison of hematological parameters between patients during remission and healthy controls

Parameter	Remission period	Control group	*p* value
WBC (×10³/µL)	7.91 (6.27–9.44)	6.90 (5.63–9.01)	0.162
Hb (g/dL)	13.35 ± 1.49	13.41 ± 1.01	0.828
RDW (%)	14.17 ± 1.24	12.79 ± 1.46	**< 0.001**
PLT (×10³/µL)	308.60 ± 70.38	276.82 ± 54.24	**0.024**
MPV (fL)	10.11 ± 1.38	7.33 ± 0.90	**< 0.001**
Neutrophil (×10³/µL)	3.87 (2.96–5.03)	3.59 (2.92–5.13)	0.961
Lymphocyte (×10³/µL)	3.02 (2.34–3.36)	2.28 (1.99–2.77)	**0.003**
Monocyte (×10³/µL)	0.50 (0.43–0.61)	0.47 (0.41–0.56)	0.313
NLR	1.29 (1.12–1.85)	1.53 (1.32–2.13)	**0.049**
PLR	110.83 (91.33–122.64)	112.18 (92.15–148.23)	0.392
PMR	589.43 (522.02–675.81)	563.62 (418.69–734.90)	0.519

*Values are presented as mean ± standard deviation for normally distributed variables and median (interquartile range) for non-normally distributed variables. Comparisons between independent groups were performed using Student’s t-test or the Mann–Whitney U test, as appropriate. Abbreviations: WBC, white blood cell count; Hb, hemoglobin; RDW, red cell distribution width; PLT, platelet count; MPV, mean platelet volume; NLR, neutrophil-to-lymphocyte ratio; PLR, platelet-to-lymphocyte ratio; PMR, platelet-to-monocyte ratio

When the active and remission periods of the same patients were compared, WBC, PLT, neutrophil count, and PMR values were significantly higher during the active phase, whereas MPV values were significantly higher during remission. No significant differences were observed in hemoglobin level, RDW, lymphocyte count, monocyte count, NLR, or PLR (Table 4)

**Table 4:** Comparison of hematological parameters between active and remission periods in the same patients with idiopathic uveitis

Parameter	Active period	Remission period	*p* value*
WBC (×10³/µL)	9.23 (7.22–10.78)	7.91 (6.27–9.44)	**0.010**
Hb (g/dL)	13.20 ± 0.84	13.35 ± 1.49	0.500
RDW (%)	13.75 ± 1.25	14.17 ± 1.24	0.082
PLT (×10³/µL)	346.40 ± 71.09	308.60 ± 70.38	**< 0.001**
MPV (fL)	9.75 ± 1.32	10.11 ± 1.38	**0.001**
Neutrophil (×10³/µL)	4.86 (3.61–5.89)	3.87 (2.96–5.03)	**0.021**
Lymphocyte (×10³/µL)	3.11 (2.32–3.65)	3.02 (2.34–3.36)	0.226
Monocyte (×10³/µL)	0.53 (0.35–0.64)	0.50 (0.43–0.61)	0.856
NLR	1.49 (1.08–2.07)	1.29 (1.12–1.85)	0.160
PLR	110.78 (94.67–133.57)	110.83 (91.33–122.64)	0.146
PMR	664.26 (565.53–879.22)	589.44 (522.02–675.81)	**0.008**

*Values are presented as mean ± standard deviation for normally distributed variables and median (interquartile range) for non-normally distributed variables. Paired Student’s t-test or the Wilcoxon signed-rank test was used for paired comparisons, as appropriate. Abbreviations: WBC, white blood cell count; Hb, hemoglobin; RDW, red cell distribution width; PLT, platelet count; MPV, mean platelet volume; NLR, neutrophil-to-lymphocyte ratio; PLR, platelet-to-lymphocyte ratio; PMR, platelet-to-monocyte ratio

In an additional subgroup analysis according to the anatomical subtype of uveitis, no statistically significant differences were observed in CBC-derived inflammatory parameters among patients with anterior, intermediate, and panuveitis (all *p* > 0.05; Supplementary Table S1)

## Discussion

In the present study, we demonstrated that several CBC-derived inflammatory parameters differed between children with idiopathic uveitis and healthy controls and also varied according to disease activity. Patients in the active phase had significantly higher WBC, RDW, platelet count, MPV, neutrophil count, lymphocyte count, and PMR values than healthy controls. Although WBC, neutrophil count, and PMR decreased during remission, RDW, platelet count, MPV, and lymphocyte counts remained elevated, suggesting persistent low-grade inflammation despite clinical remission. These findings suggest that systemic inflammatory alterations may accompany ocular inflammation even in idiopathic uveitis that appears to be clinically confined to the eye. Notably, all patients had normal CRP levels, indicating that CBC-derived parameters may identify subtle or subclinical inflammatory alterations not reflected by conventional acute-phase reactants.

Pediatric idiopathic uveitis is an uncommon but potentially vision-threatening inflammatory disease with a chronic and often insidious course [1, 8]. Currently, objective biomarkers reflecting disease activity remain limited. Therefore, CBC-derived inflammatory markers have attracted increasing interest because they are inexpensive, readily available, and routinely measured in clinical practice. However, evidence regarding CBC-derived inflammatory biomarkers in pediatric idiopathic uveitis remains limited. Most previous studies have focused on Behçet’s disease–associated uveitis or adult populations [9–13]. More recently, Ashour et al. demonstrated that the systemic immune-inflammation index (SII) was significantly higher in patients with active Behçet’s uveitis than in those with inactive disease and healthy controls, suggesting that CBC-derived inflammatory indices may provide valuable information regarding ocular inflammatory activity [14]. In addition, Akay et al. reported that pediatric noninfectious uveitis is frequently characterized by a chronic course, bilateral involvement, and a high rate of ocular complications, emphasizing the importance of close multidisciplinary evaluation and long-term follow-up in this population [4]. Although their study did not evaluate CBC-derived inflammatory biomarkers, it provides important insights into the clinical characteristics and long-term course of pediatric noninfectious uveitis. Given the limited availability of objective biomarkers for disease activity, the available evidence, together with the findings of the present study, suggests that CBC-derived inflammatory parameters warrant further investigation as readily available biomarkers for the assessment and monitoring of disease activity in pediatric idiopathic uveitis.

The observed increase in platelet count and MPV during the active phase may reflect enhanced platelet activation, which has been shown to play a role in inflammation and immune modulation. Previous studies have reported conflicting results regarding MPV in inflammatory diseases; while some authors have suggested decreased MPV as a marker of active inflammation [15, 16], others have reported increased MPV values associated with disease activity [17, 18]. In our cohort, MPV was elevated in both active and remission phases compared with controls, supporting the hypothesis that platelet activation may contribute to persistent inflammatory alterations in idiopathic uveitis.

In addition, a retrospective study in patients with idiopathic acute anterior uveitis demonstrated significantly elevated leukocyte, neutrophil, platelet count, NLR, and PLR values compared with healthy controls, supporting the presence of systemic inflammatory activation even in clinically localized ocular inflammation [19]. However, unlike our findings, MPV values did not significantly differ between groups. Moreover, in contrast to previous studies, all patients in our cohort had normal CRP levels, further supporting the concept that complete blood count–derived inflammatory markers may identify ongoing low-grade systemic inflammation even in the absence of elevated conventional acute-phase reactants.

Similarly, RDW, a marker traditionally associated with erythrocyte size variability, has also been associated with inflammation and adverse clinical outcomes in various conditions [20–22]. In our study, RDW values were significantly higher in both active and remission phases compared with controls, indicating the persistence of low-grade systemic inflammation even during clinically inactive disease. This observation aligns with findings from other inflammatory conditions, in which elevated RDW has been associated with increased disease activity and adverse clinical outcomes. These findings suggest that RDW may serve as a potentially sensitive marker of subclinical inflammation in pediatric idiopathic uveitis.

Interestingly, several parameters, including RDW, platelet count, MPV, and lymphocyte count, remained elevated during the remission phase compared with healthy controls. This observation suggests that subclinical inflammation may persist despite apparent clinical remission. A similar pattern has been reported in other chronic inflammatory conditions, where RDW has been associated with ongoing inflammatory processes and adverse clinical outcomes [22]. These findings may have important implications for disease monitoring and long-term management.

When active and remission periods were compared within the same patients, WBC, platelet count, neutrophil count, and PMR were significantly higher during the active phase, whereas MPV was higher during remission. These findings suggest that certain hematological parameters may be more sensitive to acute inflammatory activity, while others may reflect chronic or compensatory changes. Notably, PMR was associated with disease activity and may represent a potentially useful parameter for distinguishing active disease from remission.

Although NLR has been widely proposed as a marker of systemic inflammation, our results did not demonstrate a consistent association between NLR and disease activity. While a borderline significance was observed in the remission group (*p* = 0.049), NLR did not reliably differentiate between active disease and controls or between active and remission phases. These findings differ from previous studies in adult patients with idiopathic anterior uveitis, in which NLR has been reported to correlate with inflammatory markers such as CRP [23]. Similarly, Balci et al. reported significantly higher white blood cell, neutrophil, and NLR values in patients with infectious uveitis than in those with non-infectious uveitis, suggesting that NLR may also assist in the differential evaluation of uveitis [24]. In contrast, Simsek et al. reported significantly lower NLR and higher lymphocyte-to-monocyte ratio (LMR) values in patients with Fuchs uveitis syndrome compared with healthy controls, suggesting that CBC-derived inflammatory indices may vary according to the underlying subtype and pathophysiology of uveitis [25]. However, unlike these studies, our cohort consisted exclusively of children with idiopathic non-infectious uveitis, and the primary objective was to evaluate disease activity rather than etiological differentiation. Furthermore, because only patients with normal CRP levels were included, the observed alterations in several CBC-derived parameters suggest that these markers may detect low-grade or subclinical inflammation even in the absence of elevated conventional acute-phase reactants.

While CRP is widely used as an acute-phase reactant, standard CRP measurements may not be sufficiently sensitive to detect low-grade inflammation, as reflected by the use of high-sensitivity CRP (hs-CRP) in various clinical settings [26–28]. In our study, despite normal CRP levels, several complete blood count–derived parameters were significantly altered, suggesting that these markers may provide additional information regarding inflammatory activity not captured by conventional acute-phase reactants.

A major strength of this study is the inclusion of patients with CRP levels within the normal range, allowing complete blood count–derived parameters to be evaluated independently of conventional acute-phase reactants. This approach provides a unique perspective by minimizing the potential confounding effect of elevated CRP levels and highlighting the ability of these parameters to detect low-grade or subclinical inflammation [22]. Furthermore, the simultaneous assessment of multiple hematological indices, including RDW, MPV, and PMR, enabled a comprehensive evaluation of systemic inflammatory alterations in pediatric idiopathic uveitis.

This study has several limitations. First, its retrospective design and relatively small sample size may limit the generalizability of the findings. In addition, because most patients had bilateral uveitis, the findings may not be fully generalizable to children with other anatomical patterns of idiopathic uveitis. Another limitation is the lack of individual pre-disease baseline CBC measurements. Consequently, changes in hematological parameters could only be assessed between the active and remission phases rather than relative to each patient’s own baseline values. Finally, because of the exploratory nature of the study, multiple hematological parameters were evaluated without adjustment for multiple comparisons. Therefore, the findings should be interpreted with caution, and further validation in larger, prospective, multicenter studies is warranted.

## Conclusion

In conclusion, our findings suggest that complete blood count–derived parameters, including RDW, platelet-related indices, and PMR, may serve as useful markers for assessing inflammatory activity in pediatric idiopathic uveitis. Notably, these alterations were observed despite normal CRP levels, supporting the presence of low-grade or subclinical inflammation that may not be captured by conventional acute-phase reactants. The persistence of these alterations during the remission phase further reinforces the presence of ongoing subclinical inflammatory activity. These results highlight the potential value of readily available hematological parameters as complementary tools in disease evaluation. However, further prospective studies with larger cohorts are needed to validate these findings and clarify their clinical applicability.

## Supplementary Information

Below is the link to the electronic supplementary material.

Supplementary Material 1

## Data Availability

The datasets generated and/or analyzed during the current study are not publicly available due to confidentiality considerations but are available from the corresponding author on reasonable request.

## Abbreviations

CBC: Complete blood count
CRP: C-reactive protein
ESR: Erythrocyte sedimentation rate
MPV: Mean platelet volume
NLR: Neutrophil-to-lymphocyte ratio
PLR: Platelet-to-lymphocyte ratio
PMR: Platelet-to-monocyte ratio
RDW: Red cell distribution width
