# Supplementary material for: Complete blood count–derived inflammatory parameters in pediatric idiopathic uveitis
Source: BMC Ophthalmol. 2026 Sep 15;26:576. doi: 10.1186/s12886-026-05343-1 (PMC13576235; doi:10.1186/s12886-026-05343-1)
Supplement: Supplementary file 1 [file 12886_2026_5343_MOESM1_ESM.docx]

Comparison of complete blood count–derived inflammatory parameters according to the anatomical subtype of pediatric idiopathic uveitis during the active disease phase

| **Parameter** | **Anterior uveitis (n=10)** | **Intermediate uveitis (n=14)** | **Panuveitis (n=16)** | **p value** |
| --- | --- | --- | --- | --- |
| WBC (×10³/µL) | 9.41 ± 2.19 | 9.40 ± 2.74 | 8.60 ± 2.21 | 0.591 |
| Hb (g/dL) | 12.96 ± 0.91 | 13.11 ± 0.81 | 13.43 ± 0.81 | 0.337 |
| RDW (%) | 13.74 ± 1.31 | 13.46 ± 1.49 | 14.02 ± 0.99 | 0.483 |
| PLT (×10³/µL) | 354.20 ± 81.69 | 339.21 ± 64.30 | 347.81 ± 73.91 | 0.880 |
| MPV (fL) | 9.28 ± 1.64 | 9.54 ± 1.14 | 10.22 ± 1.17 | 0.164 |
| Neutrophil (×10³/µL) | 4.25 (3.76–5.96) | 5.49 (3.99–6.21) | 4.70 (3.57–5.25) | 0.419 |
| Lymphocyte (×10³/µL) | 3.63 ± 0.75 | 2.98 ± 1.19 | 2.96 ± 1.31 | 0.299 |
| Monocyte (×10³/µL) | 0.62 (0.50–0.66) | 0.55 (0.32–0.58) | 0.48 (0.35–0.62) | 0.503 |
| NLR | 1.19 (1.01–1.51) | 1.77 (1.19–2.15) | 1.57 (1.23–1.89) | 0.252 |
| PLR | 102.10 (88.02–111.11) | 112.96 (91.72–136.55) | 117.89 (100.95–154.34) | 0.180 |
| PMR | 649.05 ± 166.32 | 736.29 ± 258.94 | 729.16 ± 209.73 | 0.584 |

*Values are presented as mean ± standard deviation (SD) for normally distributed variables and median (interquartile range [IQR]) for non-normally distributed variables. Comparisons among the three anatomical subgroups were performed using one-way ANOVA or the Kruskal–Wallis test, as appropriate. A p value <0.05 was considered statistically significant. Abbreviations: WBC, white blood cell count; Hb, hemoglobin; RDW, red cell distribution width; PLT, platelet count; MPV, mean platelet volume; NLR, neutrophil-to-lymphocyte ratio; PLR, platelet-to-lymphocyte ratio; PMR, platelet-to-monocyte ratio.
